# Prevalence and factors associated with intestinal parasitic infections among food handlers working at higher public University student’s cafeterias and public food establishments in Ethiopia: a systematic review and meta-analysis

**DOI:** 10.1186/s12879-020-4884-4

**Published:** 2020-02-19

**Authors:** Birhan Alemnew, Getnet Gedefaw, Gedefaw Diress Alen, Asmamaw Demis Bizuneh

**Affiliations:** 1 Department of Medical Laboratory Sciences, College of Health Sciences, Woldia University, P.O.Box:400, Woldia, Ethiopia; 2 Department of Midwifery, College of Health Sciences, Woldia University, P.O.Box:400, Woldia, Ethiopia; 3 Department of Public Health, College of Health Sciences, Woldia University, P.O.Box:400, Woldia, Ethiopia; 4 Department of Nursing, College of Health Sciences, Woldia University, P.O.Box:400, Woldia, Ethiopia

**Keywords:** Prevalence, Intestinal parasite, Associated factors, Meta-analysis, Systematic review, Ethiopia

## Abstract

**Background:**

Intestinal infection is still an important public health problem in low-income countries. Food handlers may be infected by a wide range of enteropathogens and have been implicated in the transmission of many infections to the public. Therefore, the aim of this review was to produce the pooled prevalence and factors associated with intestinal parasitic infections among food handlers working at higher public University student’s cafeterias and public food establishments in Ethiopia.

**Methods:**

Articles published in PubMed/Medline, Hinari, Web of Science, Science Direct, and Google Scholar were used using a search strategy. Observational studies (cross-sectional) revealing the prevalence and factors associated with intestinal parasitic infections at higher public University student’s cafeterias and public food establishments were incorporated. Meta-analysis was computed using STATA version 14 statistical software. Heterogeneity of the study was assessed using Cochrane Q test statistics and I^2^ test. The pooled prevalence of the intestinal parasitic infection and associated factors among food handlers was calculated by the random-effect model.

**Results:**

Out of 138 reviewed studies, 18 studies were included to estimate the pooled prevalence of intestinal parasitic infections among food handlers in Ethiopia. All the eighteen articles were included in the analysis. This study revealed that the pooled prevalence of intestinal parasitic infections was 28.5% (95% CI: 27.4, 29.7). *E. hystolitica /E. dispar complex* 6.38 (95% Cl: 5.73, 7.04), *A.lumbricodes* 4.12 (95% Cl: 3.56, 4.67), and *G. lamblia 3.12*(95% Cl: 2.65, 3.60) were the most common intestinal parasitic infections in this study. Untrimmed fingernail 3.04 (95% CI: 2.19, 4.22), do not washing hands after defecation 2.71 (95% CI: 1.93, 3.82), do not washing hands after touching any body parts 2.41 (95% CI: 1.64, 3.56), do not made medical checkup 2.26 (95% CI: 1.57, 3.25), and do not receive food safety training 1.79 (95% CI: 1.30, 2.45) were factors significantly and positively associated with intestinal parasitic infections.

**Conclusion:**

Parasitic infections among food handlers were significantly high. Untrimmed fingernail, do not washing hands after defecation, do not washing hands after touching any body parts, do not made regular medical checkup and do not receive food safety training were factors that increase the prevalence of intestinal parasitic infections.

## Background

Food-borne infections are common public health problems, which become a significant public health issue all over the world [1]. The related problems are high in the low and middle-income countries, due to the difficulties in adopting optimal hygienic practices during food handling [2].

Intestinal parasites in Ethiopia are widespread and the loss of human life and suffering is enormous just like other low and middle-income countries. Several food-borne disease outbreaks are associated with the poor personal hygiene of people handling foodstuffs [3]. Lack of clean and safe water, high population density, lack of proper disposal of waste, noncompliance with health standards (social and individual), lack of adequate washing of vegetables, and lack of well-cooked meat lead to a high prevalence of intestinal parasites [4–6].

A lot of communicable diseases and microorganisms can enter the body through foods and cause infection. Intestinal parasites are one of the common agents to cause intestinal infection among food handlers [7]. The dominant intestinal protozoa and helminths parasites in Ethiopia are *Giardia lamblia (G.lamblia), Entamoebahistolytica/dispar (E.histolytica/dispar), Ascarislumbricoides (A.lumbricoides) and Trichuristrichuria (T.trichuria)* [8].

Studies in Ethiopia showed that the prevalence of intestinal parasitic infection among food handlers working at University student cafeteria’s and public food establishment area such as Haramaya University cafeterias (14.3%) [9], East and West Gojjam prison (61.9%) [10], Wollo University student’s cafeteria (15%) [11], Aksum Town (14.5%) [12], Jimma University Specialized Hospital (33%) [13], Addis Ababa University Students’ Cafeteria (45.3%) [7], Hawassa University (20.6%) [14], and Mekelle University student’s cafeteria (52.4%) [2]. However, the prevalence reflected in these small and fragmented studies varied widely and remained inconclusive. Besides prevalence, identifying modifiable risk factors is a critical step in identifying potential interventions. The lack of a nationwide study that determines the prevalence and factors associated with intestinal parasitic infections among food handlers working in higher public university student’s cafeterias and public food establishments is a significant gap.

Therefore, this systematic review and meta-analysis aimed to determine the pooled prevalence of intestinal parasite and associated factors among food handlers working in higher public university student cafeterias and public food establishments’ using available studies in Ethiopia. The findings from this systematic review will highlight the prevalence and factors associated with intestinal parasitic infections among food handlers working in higher public university student’s cafeterias and public food establishments’ in Ethiopia.

## Methods

### Study design and search strategies

This systematic review and meta-analysis was conducted to compute the pooled prevalence and factors associated with intestinal parasitic infections among food handlers working in higher public University student’s cafeterias and public food establishments in Ethiopia. Ethiopia is located in the eastern part of Africa with estimated population of 106,059,710 with 20.2% living in the urban area [15, 16]. The Preferred Reporting Items for Systematic Reviews and Meta-Analysis (PRISMA) checklist was used to present and report the result of the study [17]. We searched articles written in English on international databases: PubMed/ Medline, Science Direct, Web of Science Google Scholar, Hinari, and Cochrane Library [18]. Besides, Gray literature was searched through the review of available references. Besides, unpublished papers in the field of our systematic review and meta-analysis were included, online repository library including Addis Ababa, Mekelle University, Jimma University, Haramaya University, Hawassa University, and University of Gondar digital library was searched. We searched the included literature within the time interval of March 1–May1, 2019. The core search terms and phrases were “prevalence”, “intestinal parasite”, “associated factors”, “food handlers”, “University student cafeterias”, public food establishment’s” and “Ethiopia”. The search terms were used separately and in combination using Boolean operators like “OR” or “AND”.

### Inclusion and exclusion criteria

#### Inclusion criteria

Studies published until May 1, 2019.

All observational study designs (i.e., cross-sectional, case-control and cohort) reporting the prevalence of IPI among food handlers.

Both published and unpublished articles reported in the English language.

Only studies involving among food handler working at university student cafeteria’s and public food establishments in Ethiopia.

#### Exclusion criteria

Articles which were not fully accessed (full texts not available, no responding of contacting of the corresponding author via email two times).

### Outcomes of the study

The measurement outcome of this study has two main outcome variables. Intestinal parasitic infection is the primary outcome of the study whereas associated factors of IPs among food handlers working at university student cafeterias was the second outcome variable. Intestinal parasitic infection is defined as infection caused by one or more parasite [19]. The prevalence of IPIs was computed as the total number of IPIs cases divided by the total number of food handlers involving in the study multiplied by 100. The association between IPIs and associated factors were calculated in the form of the log odds ratio. The odds ratio was calculated for the common associated factors of the reported studies. The most common associated factors included in this systematic review and meta-analysis were fingernail trimming, hand washing after defecation, hand washing after touching any body parts, regular medical checkup, and food safety training.

### Data extraction

Data extraction was implemented using a standard and extraction format adapted from the JBI data extraction format. Two authors (BA and AD) were involved independently to extract all the necessary information’s from both published and unpublished studies and recorded by Microsoft excel spreadsheet. During the time of data extraction, discrepancies between two authors were resolved through discussion and consensus. This systematic review and meta-analysis included two primary outcomes. During data collection, the first outcome variable included the prevalence of IPs with 95% CI, response rate, sample size, study design, and study area. Finally, data was extracted via a Microsoft Excel spreadsheet and the log odds ratio of each associated factors were calculated.

### Quality assessment

The adopted Newcastle-Ottawa Scale (NOS) quality assesment tool was used for cross-sectional, cohort and case-control studies to assess the quality of each study. In this systematic review and meta-analysis, all the included articles were cross-sectional studies. Methodological quality, comparability and the outcome and statistical analysis of the study were the three major assessment tools that we used to declare the quality of the study. Moreover, studies scored a scale of ≥6 out of 10 was considered as having good quality. Two authors (BA and AD) independently assessed the quality of each original study using the adopted Newcastle-Ottawa Scale (NOS) quality assessment tool. During quality appraisal of the articles, any discrepancies between the two authors were handled and resolved by taking the second group authors (GG and GDA). All of the studies were included based on the adopted Newcastle-Ottawa Scale (NOS) quality assessment criteria.

### Data analysis

Random effect model was applied to estimate the pooled prevalence of IPs. After extraction of the articles in Microsoft Excel spreadsheet format, the analysis was carried out using STATA version 14 statistical software. Cochrane Q-test and *I*^2^statistics were computed to assess heterogeneity among studies [20]. After computing the statistics, results showed there is significant heterogeneity among studies (*I*^2^ = 96.80%, *p* < 0.001). To compute the overall proportion of IPs, through back-transform of the weighted mean of the transformed proportions arcsine variance weights and Dersimonian-Laird weights for fixed-effects model and random effect model respectively [21, 22]. Subgroup analysis was done based on the study setting (University vs. public food establishments) and sample size to minimize the random variations between the point estimates of the primary study. Forest plot format was used to present the pooled point prevalence with 95%Cl. For associations, a log odds ratio was used to decide the association between associated factors and IPIs among food handlers in the included studies.

## Results

**Fig. 1:**
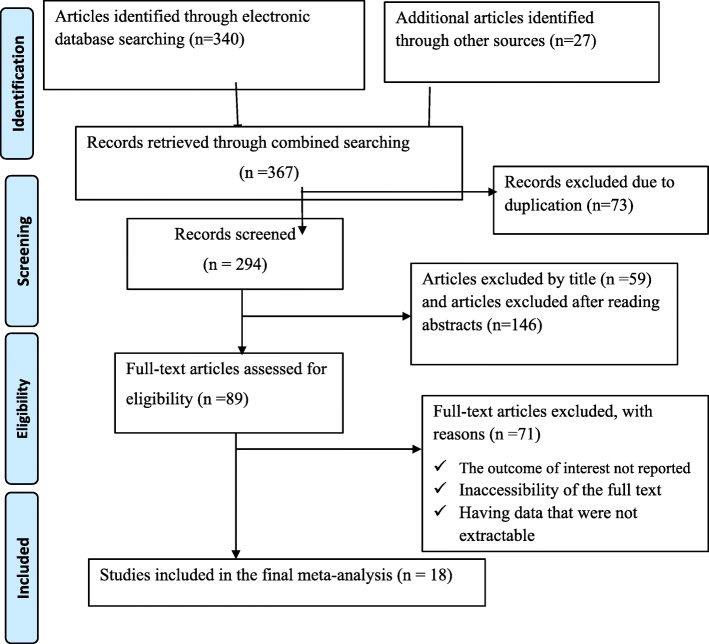
Flow chart of study selection for systematic review and meta-analysis prevalence and factors associated with intestinal parasitic infections among food handlers working in higher public university students cafeterias in Ethiopia

The electronic online search and other sources yielded 367 records regarding the prevalence and factors associated with intestinal parasitic infections among food handlers in Ethiopia, of which 73 duplicate records were identified and removed. Title and abstract screening resulted in the exclusion of 205 irrelevant articles. Therefore, 89 full-text articles were accessed and assessed for eligibility based on the pre-set criteria, which resulted in the further exclusion of 71 articles primarily due to the outcome of interest not reported, inaccessibility of the full text and having data that were not extractable. Finally, a total of 18 studies meet the eligibility criteria and were included in the meta-analysis. This systematic review and meta-analysis consist of eighteen cross sectional studies (Fig. 1).

### Characteristics of original studies

Among 18 studies which were published in Ethiopia from 2000 to 2019, 5049 study participants were involved to determine the pooled prevalence of IPIs among food handlers. Regarding the study design, almost all the studies are cross-sectional. The sample size of the studies ranged from 94 to 417. The lowest prevalence of IPIs among food handlers were reported in studies conducted in Wollo University student’s cafeteria (15%) [11], Awi Amhara (14.75%) [23], and Aksum town, Tigray (14%) [12], whereas the highest prevalence (61.78%) was reported in a study conducted at East and West Gojjam public prison [10]. Seven of the studies were from Amhara region [10, 11, 23–27], five from SNNP region [8, 14, 28–30], three from Oromia region [9, 13], one from Addis Ababa **(7)** and two from Tigray region [2, 12]. However, there were no studies reported from Benishangul Gumuz, Harari and Gambela regions, and Dire Dawa. Regarding quality score, the quality score of each original study ranged from a low of five to a high of eight (Table 1).

**Table 1:** Characteristics of studies included in the systematic review and meta-analysis

Author	Publication Year	Region	Study Area	Sample Size	Quality score	Prevalence with 95%
Solomon et al., [8]	2018	SNNP	Wolaitasodo town	387	6	40 (35, 45)
Maram et al., [9]	2018	Oromia	Haramaya University	417	6	25.2 (21, 29)
Asires et al., [10]	2019	Amhara	Debre Markos Prison	416	6	62 (57, 66)
Kebede et al., [11]	2019	Amhara	Wollo University	200	7	15 (10, 20)
Gezehegn et al., [12]	2017	Tigray	Aksum Town	400	7	15 (11, 18)
Mama et al., [31]	2016	SNNP	Arba Minch University	378	6	32 (28, 37)
Tefera et al., [28]	2014	Oromia	Yebu Town	118	5	44 (35, 53)
Andargie et al., [25]	2008	Amhara	Gondar Town	127	7	29 (21, 37)
Girma et al., [13]	2017	Oromia	JimmaUniversity	94	6	33 (23, 42)
Aklilu et al., [7]	2014	Addis Ababa	Addis Ababa University	172	7	45 (37, 53)
Abera et al., [24]	2010	Amhara	Bahir Dar Town	384	8	41 (36, 46)
Gebreyesus et al., [2]	2014	Tigray	Mekelle University	307	6	52 (47, 58)
Dagnew et al., [26]	2012	Amhara	University of Gondar	200	7	25 (19, 31)
Desta et al., [14]	2014	SNNP	Hawassa University	272	5	21 (16, 25)
Wadilo et al., [32]	2016	SNNP	Wolaitasodo town	288	5	34 (28, 39)
Alemu et al. [23]	2019	Amhara	Chagni town	400	6	15 (11,18)
Kumma et al. [30]	2019	SNNP	Wolaitasodo university	233	5	24 (18, 29)
Demis et al. [27]	2019	Amhara	Woldia university	256	7	17 (12, 21)

*SNNP* Southern Nations, Nationalities, and People

### Prevalence intestinal parasitic infections among food handlers working in higher public university student’s cafeterias and public food establishments in Ethiopia

**Fig. 2:**
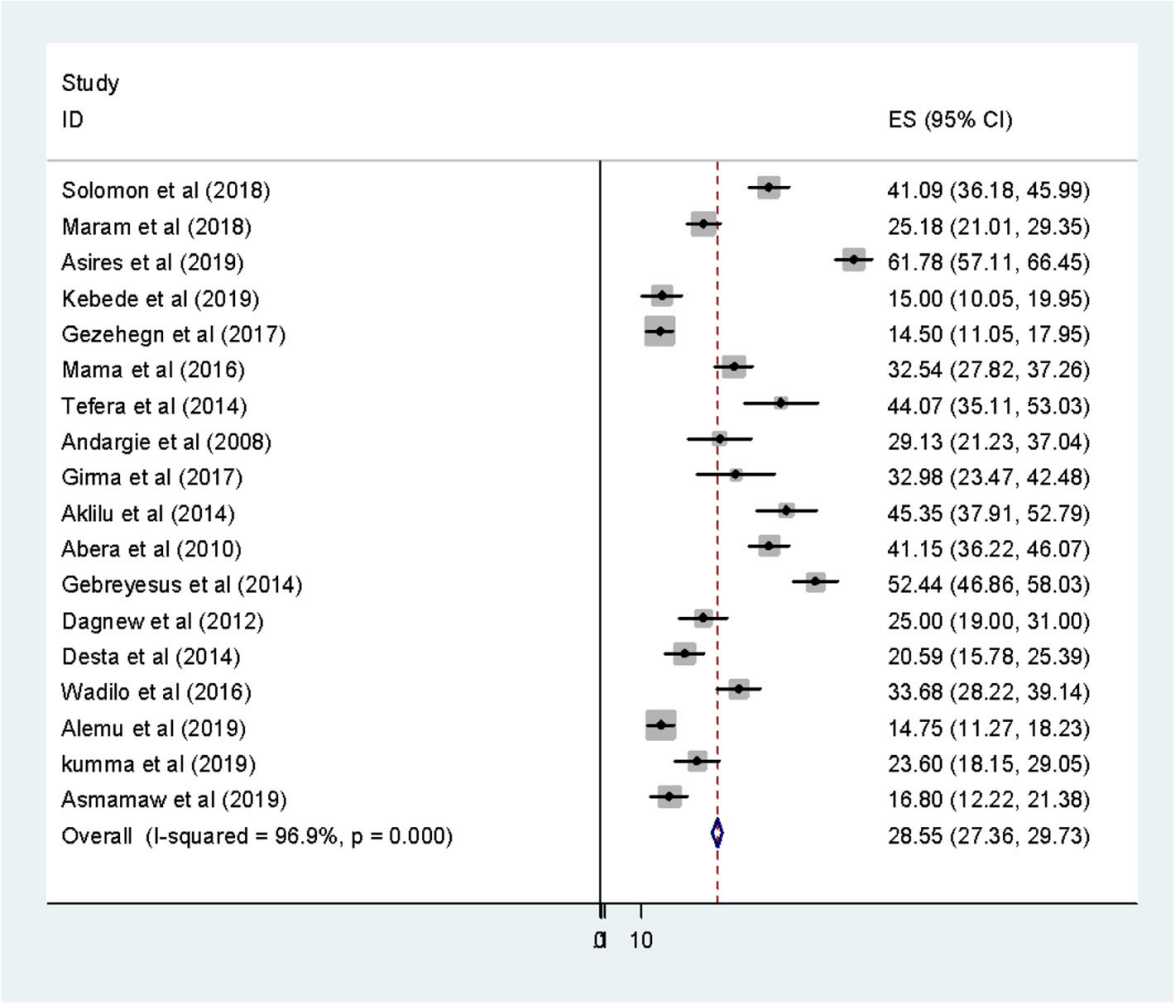
Forest plot of the pooled prevalence intestinal parasitic infections among food handlers in Ethiopia

The eighteen included studies revealed that prevalence intestinal parasitic infections among food handlers working in higher public University students cafeterias and public food establishments were 28.5% (95% CI, 27.4, 29.7) **(**Fig. 2). High heterogeneity was observed across the included studies (I^2^ = 96.90, *P* < 0.001). As a result, a random-effects model was employed to estimate the pooled prevalence of intestinal parasitic infection among food handlers in Ethiopia.

### Heterogeneity and publication bias

The existence of heterogeneity and publication bias was determined within the included studies. Consequently, there was considerable heterogeneity across fifteen included studies in this meta-analysis (I^2^ = 96.9%). Publication bias was assessed using Begg’s and Egger’s tests, showing no statistically significant for estimating the prevalence of IPIs among food handlers (*p* = 0.081) and (*P* = 0.075) respectively.

### Subgroup analysis

We performed a subgroup analysis by taking different factors. The region of the country, sample size, study area (University versus Town) was factored we considered to perform subgroup analysis. Consequently, the subgroup analysis of this study indicated that the highest prevalence of IPIs was observed in Amhara region, 27.55% (95% CI: 25.73, 29.37), SNNP, 30.39% (95% CI: 28.13, 32.64), and Oromia, 29.14% (95% Cl: 25.63, 32.65) respectively, whereas the lowest prevalence was observed in Tigray and Addis Ababa with the prevalence of 27.72% (95% CI: 24.99, 30.45) respectively (Table 3). Furthermore, subgroup analysis was performed based on the sample size of the studies. The pooled prevalence of IPIs was higher in studies having a sample size (n) > 200, 28.71% (95% CI: 27.41, 30.01) compared to those having a sample size (n) ≤2 00, 27.78% (95% CI: 24.97, 30.60). Moreover, subgroup analysis was performed based on study area/site where food handlers working in university versus public food establishments, resulting in a pooled prevalence of intestinal parasite 26.81% (95% CI: 25.14, 28.48) and 30.28% (95% CI: 28.61, 31.95), respectively (Table 2).

**Table 2:** Subgroup pooled prevalence of intestinal parasite among food handlers in Ethiopia, 2019(*n* = 18)

Variables	Characteristics	Included studies	Sample size	Prevalence with (95% CI)
Region	Amhara	7	1983	27.55 (25.73, 29.37)
	Oromia	3	629	29.14 (25.63, 32.65)
	Addis Ababa and Tigray	3	879	27.72 (24.99, 30.45)
	SNNP	5	1558	30.39 (28.13, 32.64)
Sample size	>200	12	4138	28.71 (27.41, 30.01)
	≤200	6	911	27.78 (24.97, 30.60)
Study site	University cafeterias	10	2529	26.81 (25.14, 28.48)
	Public town food establishments	8	2520	30.28 (28.61, 31.95)
Overall		18	5, 049	28.55 (27.36, 29.73)

### Common intestinal parasites among food handlers working in higher public university student’s cafeterias and public food establishments in Ethiopia

Furthermore, in this meta-analysis, the overall pooled prevalence of the common type of intestinal parasites among food handlers was observed from 18 studies as showed (Table S1). The pooled prevalence of *E. hystolitica /E. dispar complex* 6.38%(95% CI:5.73, 7.04), *A. lumbricoides 4.12*%(95% CI:3.56, 4.67), *G. lamblia 3.12*%(95% CI:2.65, 3.60), *E.vermicularis 2.69*%(95% CI:1.43,3.96), *Hookworm* 1.70%(95% CI:1.31, 2.09), *Taenia species 1*.07% (95% CI:0.75, 1.40), *H.nana* 1.03% (95% CI:0.66, 1.41), *T. trichuria* 0.84% (95% CI:0.42, 1.26), and *S. mansoni* 0.70%(95% CI:0.34, 1.07) was found from food handlers in Ethiopia (Table 3).

**Table 3:** Pooled prevalence of some common intestinal parasites among food handlers in Ethiopia

Types of intestinal parasites	Pooled prevalence (95% CI)	I-Squared
*A.lumbricoides*	*4.12* (3.56, 4.67)	95.3%, *p* < 0.001
*E. hystolitica /E. dispar complex*	6.38 (5.73, 7.04)	95.0%, *p* < 0.001
*G. lamblia*	*3.12* (2.65, 3.60)	76.8%, *p* < 0.001
*Taenia spp.*	1.07 (0.75, 1.40)	73.9%, *p* < 0.001
*Hookworms*	1.70 (1.31, 2.09)	83.1%, *p* < 0.001
*T. trichuria*	0.84 (0.42, 1.26)	12.7%, *p* = 0.33
*H. nana*	1.03 (0.66, 1.41)	90.7%, *p* < 0.001
*E. vermicularis*	*2.69* (1.43,3.96)	53.0%, *p* = 0.119
*S. mansoni*	0.70 (0.34, 1.07)	51.1%, *p* = 0.056

### Factors associated with intestinal parasitic infections among food handlers in Ethiopia

**Fig. 3:**
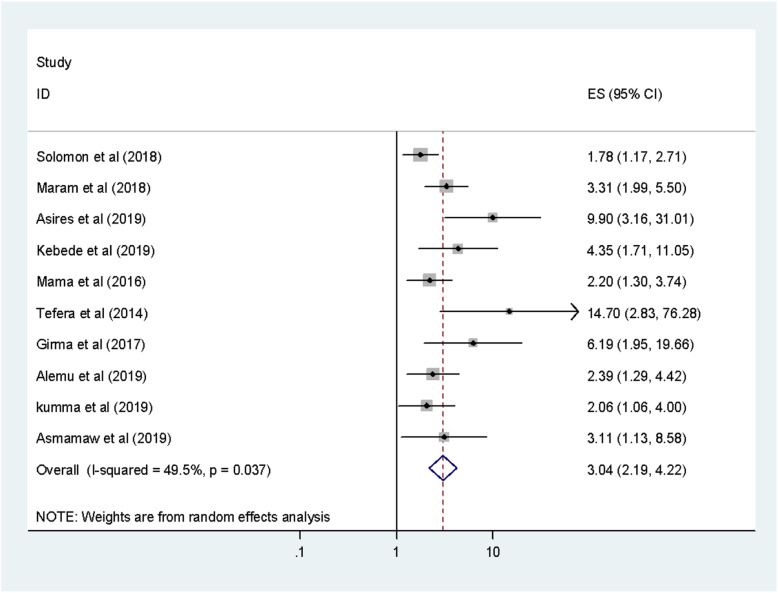
The pooled odds ratio of the association between nail trimming and IPIs among food handlers in Ethiopia

We observed the association between fingernail trimming and intestinal parasitic infections in this meta-analysis [8–11, 13, 23, 27–30]. These ten studies finding showed that the occurrence of intestinal parasitic infections was significantly associated with nail trimming habits of food handlers. Hence, the odds of intestinal parasitic infections occurrence was 3.04 times higher among food handlers who hadn’t regular nail trimming habits as compared to those who had regular nail trimming habits (OR: 3.04 95% CI: 2.19, 4.22). The finding of these test statistics revealed that there is low heterogeneity (I^2^ = 49.5% and *P* = 0.037). As a result, a random effect model was implemented to determine the association. Possibility of publication bias was detected using Begg’s and Egger’s tests with a *p*-value of 0.020 and 0.002 respectively (Fig. 3).

**Fig. 4:**
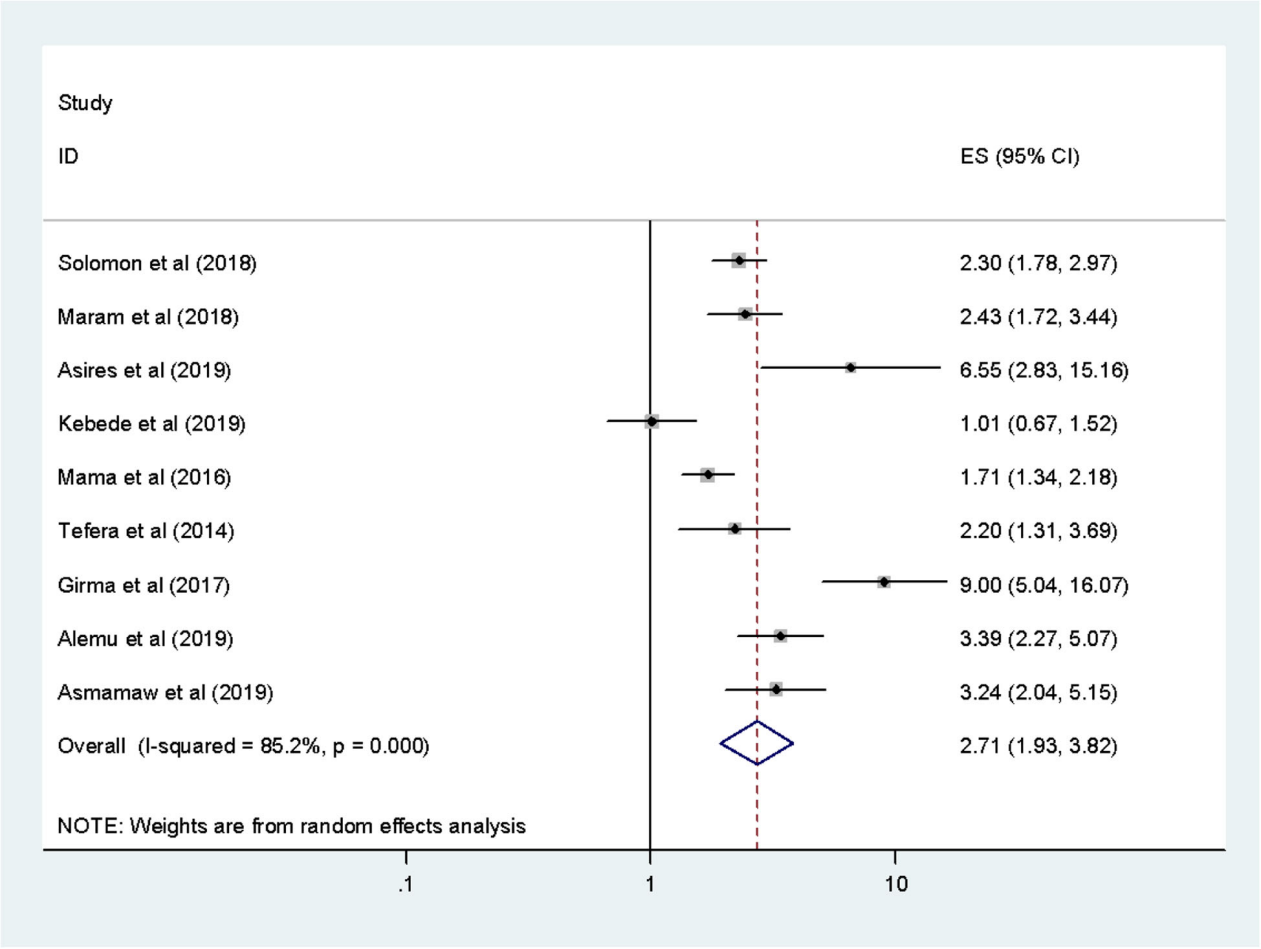
The pooled odds ratio between hands washing after defecation with intestinal parasitic infection among food handlers in Ethiopia

The association between handwashing after defecation with intestinal parasitic infections was evaluated by using seven studies [8, 10, 11, 13, 23, 25, 27–29]. This meta-analysis result revealed that hand washing after defecation is not significantly associated with intestinal parasitic infection [11]. However, the odds of having intestinal parasitic infections was 2.71 higher among food handlers who hadn’t hand washing after defecation as compared with the counterparts (OR: 2.71, 95%; CI: 1.93, 3.82) (Fig. 4). This studies showed that there was the existence of high heterogeneity (I^2^ = 85.2% and *P* < 0.001) therefore, random-effect meta-analysis was considered. No publication bias was detected using Begg’s and Egger’s tests with a *p*-value of 0.118 and 0.107 respectively.

**Fig. 5:**
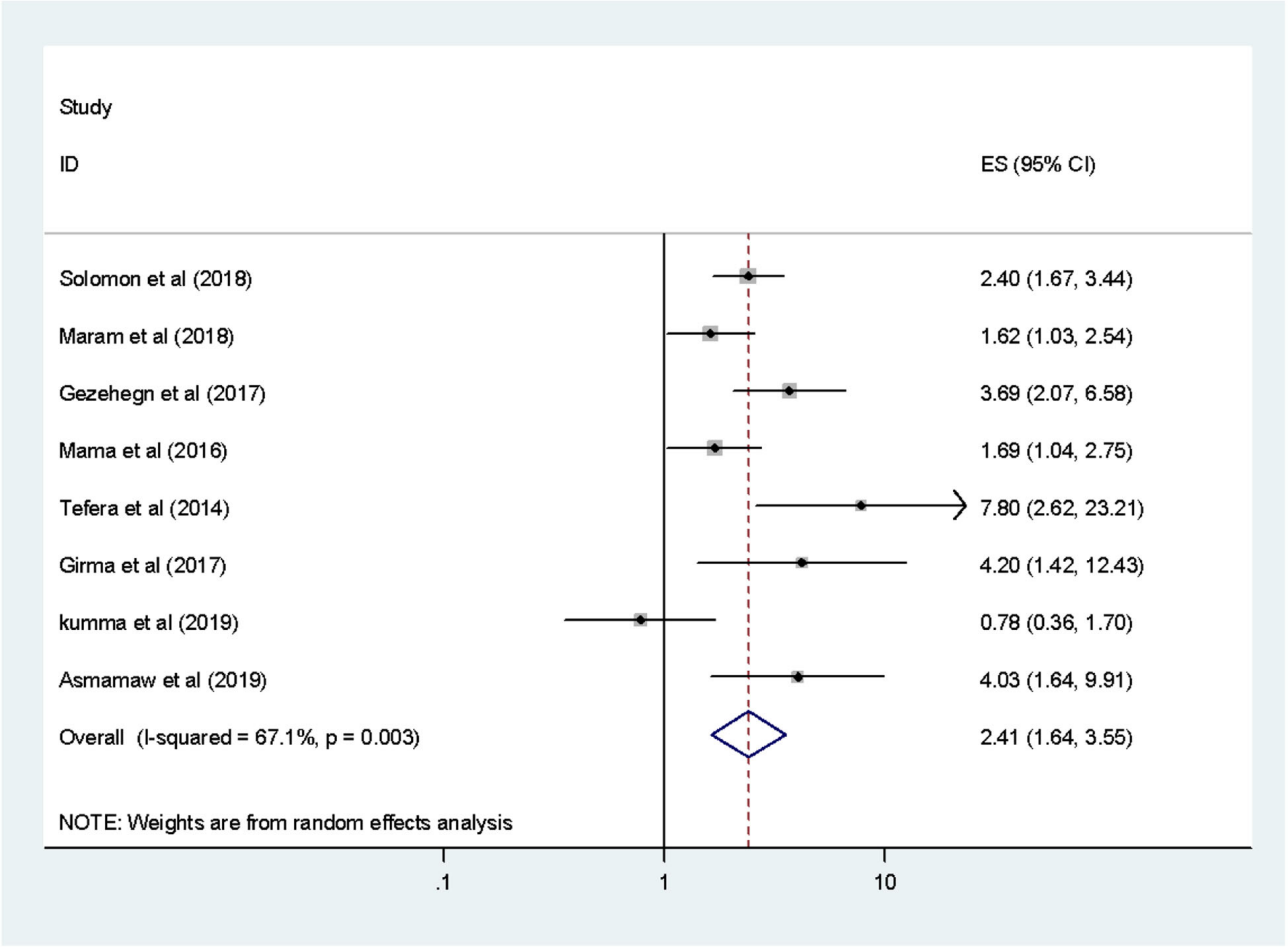
The pooled odds ratio of the association between handwashing after touching any body parts with intestinal parasitic infection among food handlers in Ethiopia

The association between handwashing after touching any body parts with intestinal parasitic infection among food handlers was computed by using eight studies [8, 9, 12, 13, 27–30]. The overall finding of this study showed that food handlers who didn’t wash their hands after touching any body parts were 2.41 higher than their counterparts (OR:2.41, 95% CI:1.64, 3.55) (Fig. 5). Moderate heterogeneity (I^2^ = 67.1%; *P*-value = 0.003) was observed among the included studies; hence, a random effect meta-analysis model was employed. Moreover, publication bias was detected using the Begg’s and Egger’s tests with a *p*-value of 0.266 and 0.376 respectively.

**Fig. 6:**
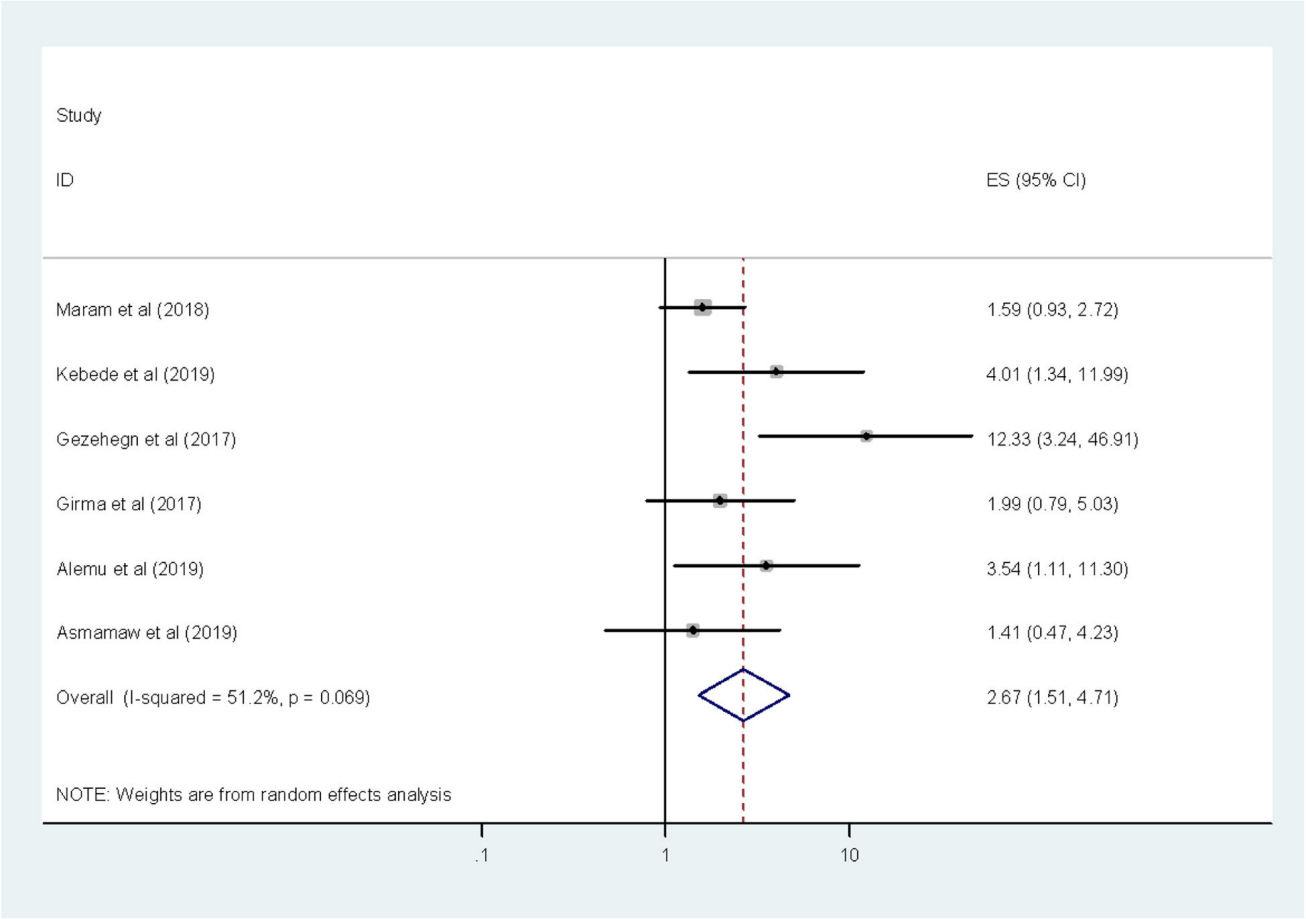
The pooled odds ratio of the association between regular medical checkup and intestinal parasitic infections among food handlers in Ethiopia

Similarly, the association between regular medical checkup and intestinal parasitic infection among food handlers in Ethiopia were computed in this meta-analysis [9, 11–13, 23, 27]. The overall meta-analysis report showed that food handlers who hadn’t regular medical checkup were 2.67 more likely to have intestinal parasitic infections than those who had regular medical checkup (OR: 2.67, 95% CI: 1.51, 4.71) (Fig. 6). Low heterogeneity (I^2^ = 51.2%; *p*-value = 0.069) was detected among the included studies; for this reason, a random effect meta-analysis model was computed. Furthermore, no possible publication bias was detected using the Begg’s and Egger’s tests with a *p*-value of 0.133 and 0.103 respectively.

**Fig. 7:**
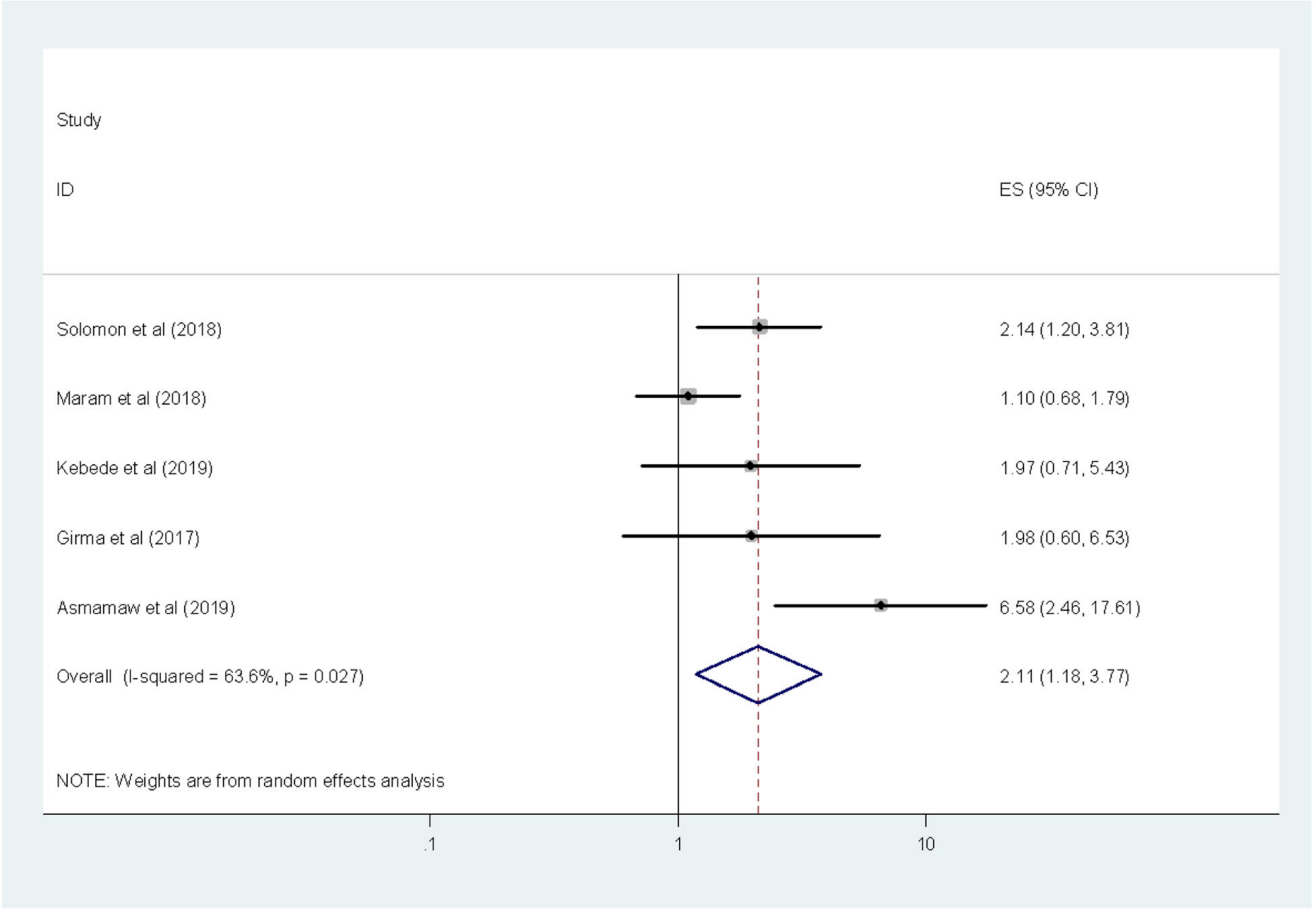
The pooled odds ratio of the association between food safety training and intestinal parasitic infection among food handlers in Ethiopia

Lastly, we employed the association between food safety training and intestinal parasitic infections. We included five studies that examined the association between intestinal parasitic infections with food safety training among food handlers [8, 9, 11, 13, 27]. The pooled result of this meta-analysis indicated that food handlers who didn’t receive food safety training were 2.11 more likely to have intestinal parasitic infections as compared with those who received food safety training (OR: 2.11, 95%CI: 1.18, 3.77) (Fig. 7). In this meta-analysis, the included studies were characterized by low heterogeneity (I^2^ = 63.6%; *P* = 0.027). Furthermore, low publication bias was detected using the Begg’s and Egger’s tests with a *p*-value of 1 and 0.248 respectively.

## Discussion

Intestinal parasitic infection is one of the most common causes of morbidity and mortality among food handlers in Ethiopia [33]. Ballpark figuring of the pooled prevalence of intestinal parasitic infection and its associated factors in Ethiopia may give attention to policymakers to take a corrective action based on the evidence. Hence, this systematic review and meta-analysis were conducted to estimate the overall pooled prevalence of IPIs and its associated factors among food handlers in Ethiopia. The overall pooled prevalence of IPIs obtained from this meta-analysis showed that (28.5%; 95% CI: 27.4, 29.7) among food handlers in Ethiopia were suffered from IPIs*.* The finding of this meta-analysis was higher than the study conducted in Iran 8.8% Southwest [34], 15.5% in Sari, Northern [35], and 10.4% Shiraz [36]. On the other hand, this study finding showed that the result is in line with the study done Northwest Ethiopia (27.7%) IPIs from clinically suspected patients [37]. The possible justification for the above disparity could be credited to methodological variation in the assessment of prevalence. The discrepancy in the prevalence of IPIs this review and meta-analysis study and other African countries could be rationalized by the dissimilarity in socio-demographics, personal and environmental hygiene practice.

The subgroup analysis of this study showed that the highest prevalence of Intestinal parasitic infection was observed in SNNP, 30.39% (95% CI: 28.13, 32.64), Oromia, 29.14% (95% Cl: 25.63, 32.65) and Amhara region, 27.55% (95% CI: 25.73, 29.37), whereas the lowest prevalence was observed in Tigray and Addis Ababa with the prevalence of 27.72% (95% CI: 24.99, 30.45) respectively. The possible explanation for this variations across the region might be due to sociodemographic, environmental, behavioural characteristics of food handlers and quality of food establishments.

In this meta-analysis, the pooled prevalence of intestinal protozoa infection was *E.histolytica* 6.38% (5.73, 7.04), and *G. Lamblia 3.12*% (2.65, 3.60). The finding of this meta-analysis in line with the study done in Iran *G. lamblia* (4.52%), and *E. hystolitica /E. dispar complex* (1.39%) [34]. However, our study finding was lower than the study done in Libya and sari Northern Iran, *E. hystolitica /E. dispar complex* 19.9% *G. lamblia* 4.6% [38], and in *Giardia lamblia* (1.6%) respectively [35].

In this meta-analysis *Taenia species* 1.07% (0.75, 1.40), *Hookworm* 1.70% (1.31, 2.09), *T. trichuria* 0.84% (0.42, 1.26), *H.nana* 1.09% (0.66, 1.41), *E. vermicularis* 2.69% (1.43, 3.96), and *S. mansoni* 0.70% (0.34, 1.07) were the commonest protozoan infections. The finding of this study was relatively consistency with studies in Southwest Iran*, H.nana* (1.29%), *A.lumbricoides* (0.57%), and *E.vermicularis, T. trichiura, S. stercoralis* was each less than 0.5% [34]. However, a study conducted in Sari, Northern Iran showed that *H. nana* (1.9%) was the only helminthic infection [35]. Furthermore, a study from clinically suspected patients in northwest Ethiopia was *hookworm* 21.1%, and *A.lumbricoids* 3.9% [37]. Besides, a survey study in Ethiopia reported that *A .lumbricoides 9*.9%, *hookworm* 9.7%, and *T.trichiura* 2.6% were the commonest intestinal helminths [39].

Under subgroup analysis, the result of this study showed that the highest prevalence of IPIs was observed in SNNP, 30.73% (95% CI: 28.34, 33.11), followed by Oromia 28.81% (95% CI: 25.69, 31.94), then Tigray and Addis Ababa 27.72%(95% CI: 24.99, 30.45) whereas Amhara region was the lowest prevalent region with the prevalence of 27.55% (95% CI: 25.73, 29.37). The potential justification for this difference might be due to the distinction in socio-demographic, environmental, geographical and behavioural characteristics.

This systematic review and meta-analysis study is aimed to identify factors associated with intestinal parasitic infection among food handlers in Ethiopia. In this study, fingernail trimming, hand washing after defecation, hand washing after touching any body parts, regular medical checkup, and food safety training were significantly associated with intestinal parasitic infections.

The odds of not having handwashing after defecation were 2.71 times more likely to develop intestinal parasitic infections than their counterparts. This finding is supported by the studies conducted in Kenya [40], Gambia [41], Pakistan [42] and Ethiopia [30, 43, 44]. This might be due to feco-oral microorganism transmissions. The odds of untrimmed fingernail were 3.04 times more likely to acquire intestinal parasitic infections than their counterparts. This finding is supported by the studies conducted in Ethiopia [30, 43, 44]. This might be due to the fact that untrimmed fingernail may contain dust particles and microorganisms which facilitates in easily transmissions of microorganisms feco-orally.

The odds of didn’t take food safety training were 1.79 times more likely to be infected with intestinal parasites than those food handlers who took food safety training. This finding supported by the studies conducted in Bangladesh [45] and Saud Arabia [46]. This might be due to the fact those food handlers who didn’t take food safety training may lack the necessary knowledge and practice towards transmission and prevention of microorganisms.

## Limitations of the study

Like other meta-analyses, this study shares its own limitation. Even though the finding of published articles other than English is consistent with others but we only considered articles published only in English languages. Besides, all of the studies included in this review were cross-sectional; as a result, the outcome variable might be affected by other confounding variables.

## Conclusion

In this study, intestinal parasitic infection among food handlers in Ethiopia was significantly high. Untrimmed fingernail, do not washing hands after defecation, do not washing hands after touching any body parts, do not made regular medical checkup and do not receive food safety training were factors that increase the prevalence of intestinal parasitic infections. Therefore, based on the study findings, the authors recommend particular emphasis shall be given to the periodic and regular medical care, health educations about personal hygiene, hand washing practice, and food safety are recommended for food handlers.

### Supplementary information

**Additional file 1: Table S1.** some common intestinal parasite among food handlers from each individual study.

## Data Availability

All related data has been presented within the manuscript. The dataset supporting the conclusions of this article is available from the authors on request.

## Abbreviations

CI: Confidence Interval
IPs: Intestinal parasites
OR: Odd Ratio
PRISMA: Preferred Reporting Items for Systematic Reviews and Meta-Analysis
SNNPR: South Nation Nationalities and Peoples Region
