# Correction: Prevalence and factors associated with intestinal parasitic infections among food handlers working at higher public University student’s cafeterias and public food establishments in Ethiopia: a systematic review and meta-analysis

**DOI:** 10.1186/s12879-023-08515-2

**Published:** 2023-09-15

**Authors:** Birhan Alemnew, Getnet Gedefaw, Gedefaw Diress Alen, Asmamaw Demis Bizuneh

**Affiliations:** 1 Department of Medical Laboratory Sciences, College of Health Sciences, Woldia University, P.O.Box:400, Woldia, Ethiopia; 2 Department of Midwifery, College of Health Sciences, Woldia University, P.O.Box:400, Woldia, Ethiopia; 3 Department of Public Health, College of Health Sciences, Woldia University, P.O.Box:400, Woldia, Ethiopia; 4 Department of Nursing, College of Health Sciences, Woldia University, P.O.Box:400, Woldia, Ethiopia

**Correction: BMC Infect Dis 20, 156 (2020)**

**https://doi.org/10.1186/s12879-020-4884-4**

The original publication of this article [1] contained an incorrect author name. The incorrect and correct information is listed in this correction article.

Incorrect

- Gedefaw Diress

Correct

- Gedefaw Diress Alen

The original article has been updated to correct this error.

## References

[1] Alemnew B (2020). Prevalence and factors associated with intestinal parasitic infections among food handlers working at higher public University student’s cafeterias and public food establishments in Ethiopia: a systematic review and meta-analysis. BMC Infect Dis.

