# Supplementary material for: Prevalence and factors associated with intestinal parasitic infections among food handlers working at higher public University student’s cafeterias and public food establishments in Ethiopia: a systematic review and meta-analysis
Source: BMC Infect Dis. 2020 Feb 19;20:156. doi: 10.1186/s12879-020-4884-4 (PMC7031974; doi:10.1186/s12879-020-4884-4)
Supplement: Supplementary file 1 — Additional file 1: Table S1. some common intestinal parasite among food handlers from each individual study. [file 12879_2020_4884_MOESM1_ESM.docx]

Table S1: some common intestinal parasite among food handlers from each individual study

| Author | Year | sample size | A. lumbricoides | E.histolytica | G. lamblia | Taenia spp. | Hook  Worms | T. trichuria | H. nana | E. vermicularis | S. mansoni |
| --- | --- | --- | --- | --- | --- | --- | --- | --- | --- | --- | --- |
| Solomon et al,.(8) | 2018 | 387 | 30(7.8%) | 19(4.9%) | 21(5.4%) | 26(6.7%) | 23(5.9%) | - | - | - | - |
| Maram et al,.(9) | 2018 | 417 | 15(3.6%) | 49(11.7%) | 14(3.36%) | 5(1.19%) | 8(1.9%) | - | 11(2.64%) | - | - |
| Asires et al,.(10) | 2019 | 416 | 157 (37.7%) | 83 (19%) | 13 (3.13%) | - | 28 (6.71%) | - | 48(11.53%) | 17 (4.08% | - |
| Kebede et al,.(11) | 2019 | 200 | 8(4%) | 11(5.5%) | 6(3%) | 3(1.5%) | - | - | - | - | - |
| Gezehegn et al,.(12) | 2017 | 400 | - | 13(3.3%) | 20(5%) | - | 4(1%) | - | 10(2.5%) | - | 10(2.5%) |
| Mama et al,.(30) | 2016 | 378 | 31(8.2%) | 48(12.7%) | 18(4.7%) | 14(3.7%) | 3(0.79%) | 4(1.1%) | - | - | - |
| Tefera et al,.(27) | 2014 | 118 | 21(17.8%) | 3(2.5%) | 7(5.9%) | - | 11(9.3%) | 7(5.9%) | - | 3(2.5%) | - |
| Andargie et al,.(24) | 2008 | 127 | 23(18.11%) | 2(1.6%) | 1(0.8%) | - | 1(0.8%) | 2(1.6%) | - | - | 1(0.8%) |
| Girma et al,.(13) | 2017 | 94 | 15(16%) | 4(4.3%) | 2(2.1%) | 2(2.1%) | 1(1.1%) | 2(2.1%) | 2(2.1%) | 1(1.1%) | - |
| Aklilu et al,.(7) | 2014 | 172 | 2(1.16%) | 68(39.8%) | 18(10.46%) | 5(2.9%) | 2(1.1%) | 1(0.58%) | - | - | - |
| Abera et al,.(23) | 2010 | 384 | 45(11.7%) | 49(12.79%) | 27(7%) | 5(1.3%) | 31(8.1%) | 2(1.16%) | 2(0.52%) | - | 7(1.8%) |
| Gebreyesus et al,.(2) | 2014 | 307 | 4(1.3%) | 99(32.3%) | 15(4.9%) | 4(1.3%) | 4(1.3%) | - | 2(0.7%) | - | 1(0.3%) |
| Dagnew et al,.(25) | 2012 | 200 | 13(6.5%) | 12(6%) | 22(11%) | 1(0.5%) | - | - | - | - | 1(0.5%) |
| Desta et al,.(14) | 2014 | 272 | 26(9.5%) | 6(2.2%) | 3(1.1%) | 1(0.4%) | - | 2(0.7%) | - | - | 5(1.8%) |
| Wadilo et al,.(31) | 2016 | 288 | 18(6.25%) | 13(4.51%) | 10(3.47%) | 14(4.86%) | 17(5.9%) | 3(1.04%) | 7(2.43%) | - | 1(0.4%) |
| Alemu et al(22) | 2019 | 400 | 11(2.75%) | 34(8.5%) | 6(1.5%) | 2(0.5%) | 5(1.25%) | - | 1(0.4) | - | - |
| kumma et al(29) | 2019 | 233 | 3(1.3%) | 39(16.79) | 12(5.2) | - | 3(1.3%) | - | - | - | - |
| Demis et al(26) | 2019 | 256 | 9(3.5%) | 14(5.5%) | 10(3.9%) | 2(0.8%) | - | - | - | - | - |
